# Biodegradation of [D-Leu^1^] microcystin-LR by a bacterium isolated from sediment of Patos Lagoon estuary, Brazil

**DOI:** 10.1186/s40409-015-0001-3

**Published:** 2015-02-24

**Authors:** Gilmar AF Lemes, Luiza W Kist, Mauricio R Bogo, João S Yunes

**Affiliations:** Laboratório de Cianobactérias e Ficotoxinas, Instituto de Oceanografia, Universidade Federal do Rio Grande (FURG), Av. Itália, km 8, Campus Carreiros, Caixa postal 474, Rio Grande, RS CEP 96203-000 Brazil; Laboratory of Genomics and Molecular Biology, School of Biosciences, Pontifical Catholic University of Rio Grande do Sul (PUCRS), Porto Alegre, Rio Grande do Sul State Brazil; National Institute of Science and Technology for Translational Medicine, Porto Alegre, Rio Grande do Sul State Brazil

**Keywords:** Biodegradation, Microcystin, Sediment, Patos Lagoon, Estuary, Brazil

## Abstract

**Background:**

Toxic cyanobacterial blooms are recurrent in Patos Lagoon, in southern Brazil. Among cyanotoxins, [D-Leu^1^] microcystin-LR is the predominant variant whose natural cycle involves water and sediment compartments. This study aimed to identify and isolate from sediment a bacterial strain capable of growing on [D-Leu^1^] microcystin-LR. Sediment and water samples were collected at two distinct aquatic spots: close to the Oceanographic Museum (P1), in Rio Grande City, and on São Lourenço Beach (P2), in São Lourenço do Sul City, southern Brazil.

**Methods:**

[D-Leu^1^] microcystin-LR was isolated and purified from batch cultures of *Microcystis aeruginosa* strain RST9501. Samples of water and sediment from Rio Grande and São Lourenço do Sul were collected. Bacteria from the samples were allowed to grow in flasks containing solely [D-Leu^1^] microcystin-LR. This strain named DMSX was isolated on agar MSM with 8 g L^−1^ glucose and further purified on a cyanotoxin basis growth. Microcystin concentration was obtained by using the ELISA immunoassay for microcystins whereas bacterial count was performed by epifluorescence microscopy. The genus *Pseudomonas* was identified by DNA techniques.

**Results:**

Although several bacterial strains were isolated from the samples, only one, DMXS, was capable of growing on [D-Leu^1^] microcystin-LR. The phylogenetic analysis of the 16S rRNA gene from DMXS strain classified the organism as *Pseudomonas aeruginosa*. DMXS strain incubated with [D-Leu^1^] microcystin-LR lowered the amount of toxin from 1 μg.L^−1^ to < 0.05 μg.L^−1^. Besides, an increase in the bacterial count–from 71 × 10^5^ bacteria.mL^−1^ to 117 × 10^5^ bacteria.mL^−1^–was observed along the incubation.

**Conclusions:**

The use of bacteria isolated from sediment for technological applications to remove toxic compounds is viable. Studies have shown that sediment plays an important role as a source of bacteria capable of degrading cyanobacterial toxins. This is the first Brazilian report on a bacterium–of the genus *Pseudomonas*–that can degrade [D-Leu^1^] microcystin-LR, the most frequent microcystin variant in Brazilian freshwaters.

## Background

Cyanobacteria are also known as blue-green algae, because they have characteristics of both algae and bacteria, although they are now classified as bacteria. These organisms are capable of producing toxins, called cyanotoxins, and their blue-green color comes from the pigments which gives their ability to photosynthesize. In waterbodies used for water supply, cyanobacterial blooms can pose serious threats to animals and humans.

Toxic cyanobacterial blooms have been reported worldwide [1-7]. A serious case of cyanobacterial contamination occurred in Brazil. A hemodialysis clinic in the city of Caruaru, in northeastern Pernambuco, used water contaminated with cyanotoxins, which led to more than 60 patient deaths [8,9].

**Figure 1:**
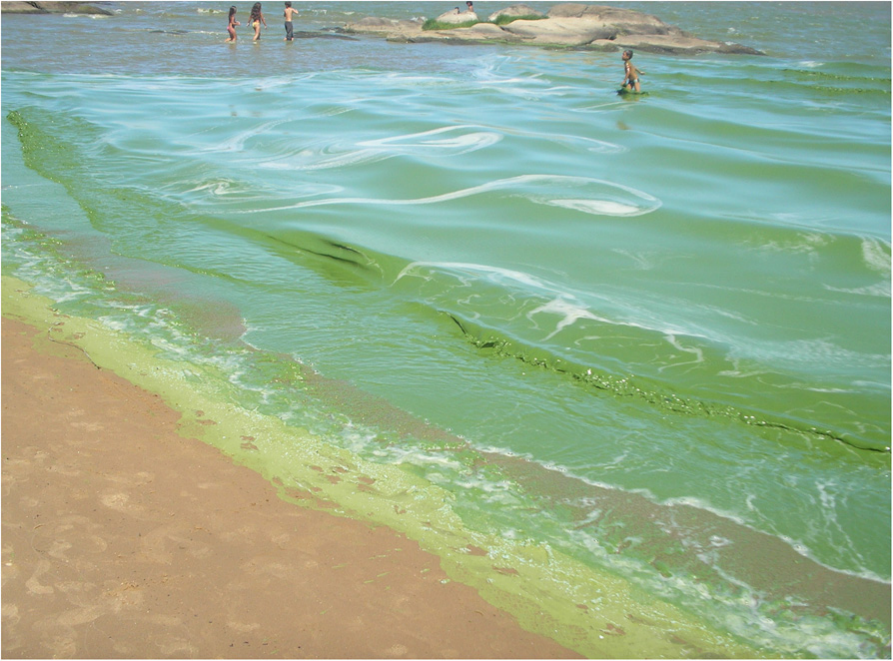
***Microcystis aeruginosa*** **bloom at São Lourenço do Sul Beach during the summer of 2005.**

In Patos Lagoon, in the southernmost part of Brazil, toxic cyanobacterial blooms have been observed for 18 years [4,10] as seen in Figure 1.

The Patos Lagoon is a large waterbody that covers an area of 10,360 km^2^. Since several freshwater rivers run into it, it involves an even higher surface area of 200,000 km^2^ in the Rio Grande do Sul state. The estuarine area comprises 10% of the lagoon and is fundamental to fish species, crustaceans and mollusks, supplying food, shelter and reproduction sites (several species come to the estuary to spawn). The economic activities of the area lies on fishery, waterways and recreational places [4,11]. Therefore, toxic cyanobacterial blooms affect not only estuarine organisms, but also the local economy.

Cyanobacterial blooms in Patos Lagoon are extremely toxic since mouse bioassays detected lethal concentrations below 100 μg.kg^−1^ of body weight [12]. These blooms are associated with pH (7.5-8.0), temperatures (>20°C) and high concentration of nutrients [4]. Among the toxins present in cyanobacterial blooms are microcystins and cyclic heptapeptides that have more than 65 structural variants with variable toxicity. Microcystins may trigger tumor development and have been associated with acute and chronic health problems in humans and other animals.

*Microcystis aeruginosa* is the main toxic species found in Patos Lagoon and is known for producing [D-Leu^1^] microcystin-LR [7,13]. This variant comprises 90% of the total amount of toxins produced intracellularly and is one of the toxins with the highest toxicity [13-15]. Investigation on the biodegradation of cyanobacterial toxins have been carried out in laboratories and in fieldwork aiming at avoiding damage caused by these blooms [16-19].

In the south of Brazil, the first study on the biodegradation of [D-Leu^1^] microcystin-LR was performed with samples of Patos Lagoon water [20]. Afterwards, another research was conducted to identify a specific strain that is capable of degrading microcystin-LR and [D-Leu^1^] microcystin-LR originated from the estuary of Patos Lagoon [11].

Since several studies recommend the biodegradation of cyanobacterial toxins as a safe and viable manner to remove toxic compounds from water, the current work aimed to isolate and identify a bacterial strain collected from the sediment of Patos Lagoon which is able to degrade the toxin [D-Leu^1^] microcystin-LR [18,21,22].

## Methods

### Cultures

Experiments were carried out with the toxin extracted from cultures of the cyanobacterium *Microcystis aeruginosa* RST9501. The strain was isolated from water collected in Patos Lagoon and deposited in the Culture Collection of the Laboratory of Cyanobacteria and Phycotoxins. Cultures were maintained in 1 L Erlenmeyer flasks with BG-11 medium [23]. Culture flasks were kept in FANEM 347 growth chambers at 20°C ± 2°C in light–dark cycles of 12 hours. The cell growth was performed in order to yield a major variant of microcystin, [D-Leu^1^] microcystin-LR, for use in the experiments.

### Extraction and purification of microcystin

The toxin [D-Leu^1^] microcystin-LR was extracted from cells according to Lawton *et al.* [24]. Cultures were centrifuged (Hermle Z323, Labnet, Germany) at 8,000 *g* for 20 minutes. Afterwards, the centrifuged material was frozen and lyophilized (Edwards MicroModulyo, UK). Then, 250 mL of acetic acid and distilled water (5% v/v) was added for each gram of lyophilized material. The mixture was agitated for an hour and again centrifuged at 8,000 *g* for ten minutes. The supernatant (S_1_) was stored. The resulting material (pellet) of this centrifugation went through a second centrifugation process that was similar to the first one. After the centrifugation at 8,000 *g* for ten minutes, the pellet was discharged and the second supernatant (S_2_) was added to the previous (S_1_). Both resulting supernatants (S_1_ + S_2 _= 500 mL) were stored in a freezer for 24 hours. After thawing, they were centrifuged at 8,000 *g* for ten minutes and the resulting pellet was discharged.

**Figure 2:**
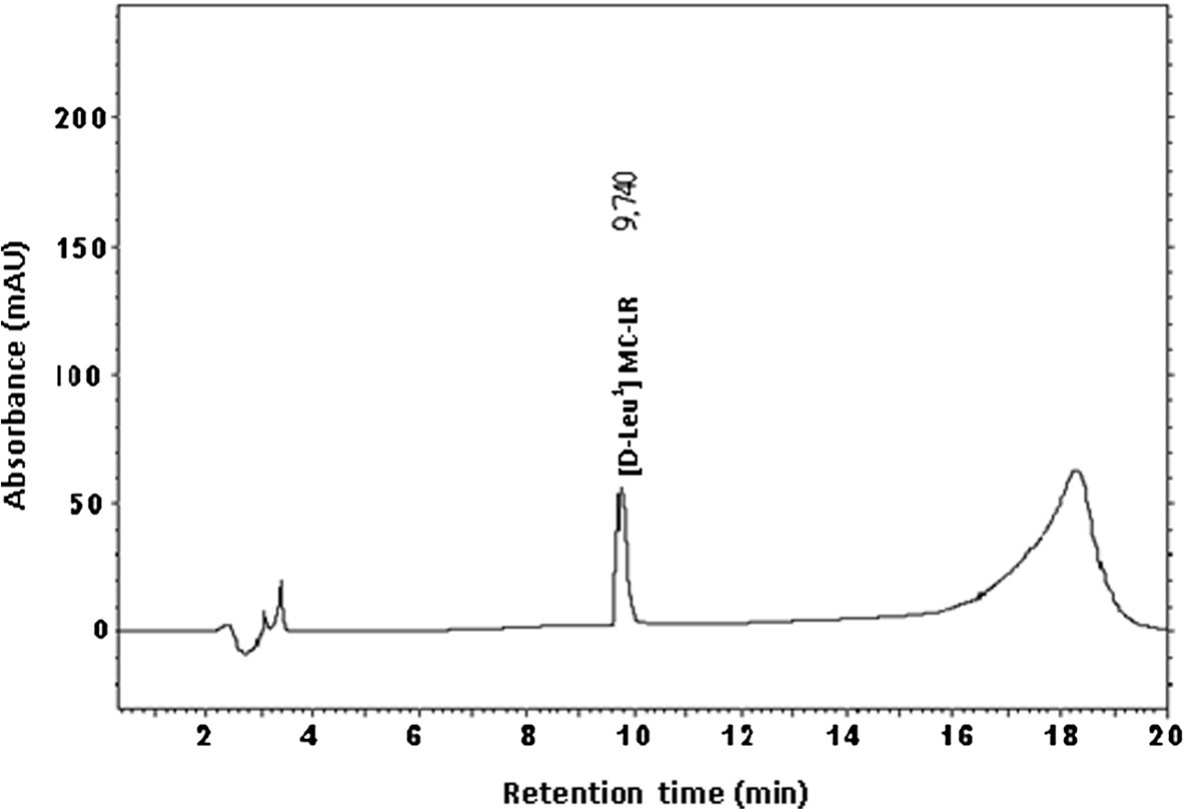
**Chromatogram of the extraction of strain RST9501 after purification.**

In the second step, total resultant supernatants were filtered through a peristaltic pump (Millipore Corporation, USA) which was coupled to a Sep-Pak 3 mL/1 g C_18_ cartridge. The cartridge was previously activated by addition of 10 mL of methanol, followed by 10 mL of distilled water. Afterwards, the supernatants were filtered through the cartridges; these containers were frozen for 24 hours. After thawing, the cartridges were eluted with 20 mL of methanol (100%). The extracts that resulted from the passage of methanol through the cartridges were dried in a rotary evaporator with a vacuum system at 40°C. After evaporation, residues were resuspended in 500 μL of methanol (100%) twice and were then analyzed by high performance liquid chromatography (HPLC–Shimadzu SCL-10A_vp_, Japan) to determine the concentration of microcystins. The resulting toxin (Figure 2) was used in the biodegradation experiments.

### Water and sediment collection

**Figure 3:**
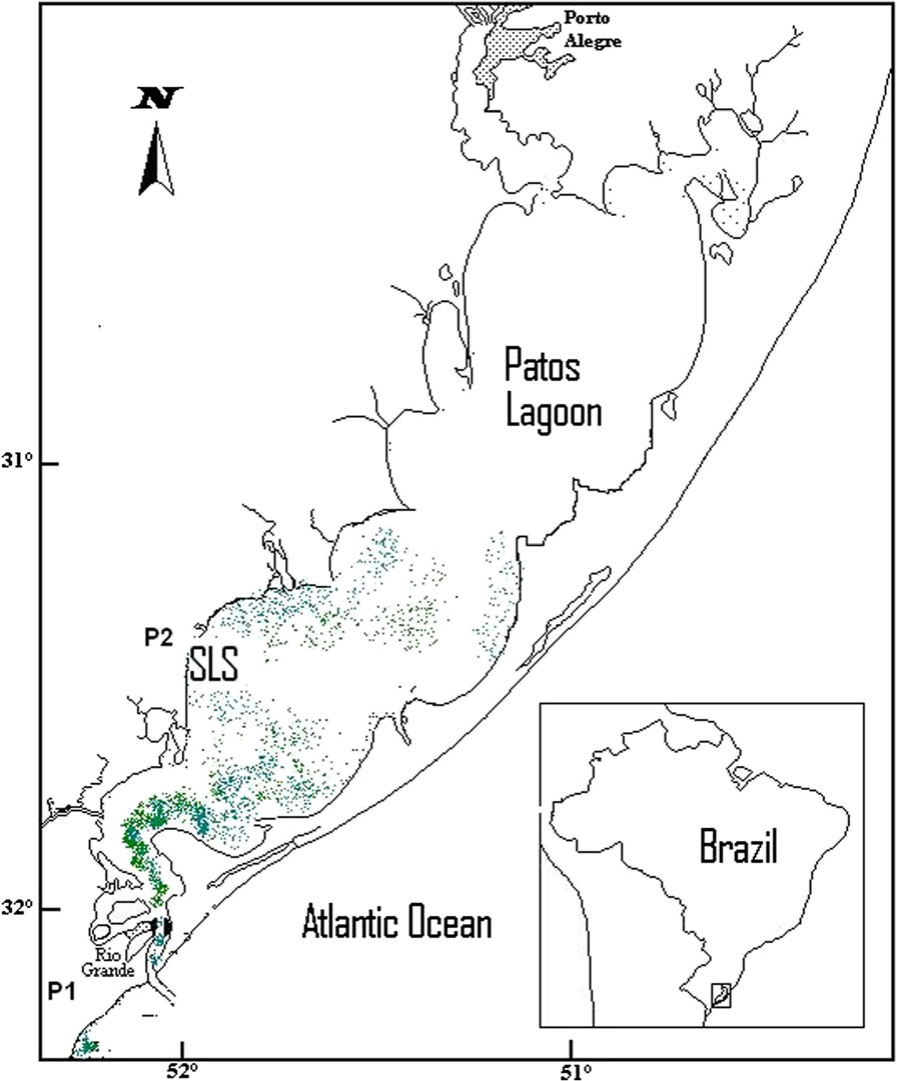
**Map of Patos Lagoon estuarine area with collection spots P1 and P2 (Brazil).**

In Patos estuary, water and sediment samples were collected at spot P1, near the Oceanographic Museum (32.0246S; 52.1062 W) that belongs to the Federal University of Rio Grande (FURG). This spot was chosen because bacteria able to degrade cyanotoxins had already been found in that environment [11]. In São Lourenço do Sul city (31.3690 S; 51.9620 W), water samples were collected on the beach, at spot P2 (Figure 3), a place where heavy *Microcystis* blooms were seen, so they also could be the source of heterotrophic bacteria.

Surface water samples were collected in a 1-L plastic bucket and stored in clean plastic bottles. Temperature (Incoterm thermometer, Brazil) and salinity (Quimis salinometer, Brazil) were measured at the site whereas the pH was measured in the laboratory (Digimed pH meter, Brazil).

Sediment was only collected at P1, near the Oceanographic Museum, about 500 g with PVC pipes (75 cm in diameter) stored in clean plastic bottles and taken to the laboratory.

Afterwards, water and sediment samples were stored in styrofoam boxes and taken to the laboratory, where they were disposed in 250 mL minimal salt media (MSM) and vortexed for 30 minutes. All samples were collected and analyzed in triplicate.

### Isolation of bacterial strains

To isolate bacterial strains, water samples were filtered through Whatman filters (7.0 cm, pore 0.45 μm), and, then, spread on Petri dishes with MSM and addition of 8 g.L^−1^ glucose anhydrous and 15 g L^−1^ agar [25]. Sediment samples were centrifuged (Hermle Z323, Labnet, Germany) at 12,000 rpm for ten minutes and the supernatant was filtered in Whatman cellulose acetate membrane filters (7.0 cm, pore 0.45 μm). The remaining pellet was discharged. Several strains were isolated from the water collected in both spots and from the sediment collected at P2, strains were catalogued and kept in cultures for the biodegradation tests (Table 1).

**Table 1:** **Bacteria isolated from water samples collected at two spots in Patos Lagoon: P1, in the Patos Lagoon estuary (near the Oceanographic Museum) and P2, on São Lourenço Beach (Rio Grande do Sul state, Brazil)**

**Bacterial strain**	**Spot**	**Color**	**Source**
BM12	P1	Yellow	Water
EE1	P1	White	Water
EBDE_1_Br	P2	White	Water
LPML	P1	Orange	Water
NOT13	P1	Yellow	Water
CEV	P1	Red	Water
BB0412	P2	Yellow	Water
FB0607	P1	Red	Water
DMXS	P1	Light brown	Sediment

### Biodegradation experiment of [D-Leu^1^] microcystin-LR

The biodegradation experiment of [D-Leu^1^] Microcystin–LR was carried out in a first screening basis, with each strain that was isolated from water and sediment samples collected at spots P1 and P2.

The experiment was conducted with 30 mL MSM in each flask, which had previously been autoclaved at 120 kgf/cm^2^ for 30 minutes. Three amber flasks were kept for the treatment and three were kept for control. Eight microliters of [D-Leu^1^] microcystin-LR at 3.7 μg.L^−1^ was added to each flask, so that the approximate concentration was 1 μg.L^−1^.

Treatment flasks had bacterial samples from the culture dishes whereas control flasks did not have bacterial strains.

The experiment was kept in a microbiological heater Certomat® BS-1 (Germany) at 27°C, in dim light to prevent the toxin from degrading due to intense light.

During the experiment, samples were collected for analysis every three days, on average; all handling was done in a microbiological chamber with a Bunsen burner. Total sampling time was 30 days. Medium samples were labeled and stored in a freezer at –20°C for further analysis by ELISA immunoassay.

### Selection and isolation of the strain to perform the biodegradation assay

At the end of the experiment with sediment, when the concentration of the toxin [D-Leu^1^] microcystin-LR reached “zero”, a sample of this bacterial strain was removed from the flasks and inoculated in MSM on a Petri dish with the addition of 8 g.L^−1^ glucose anhydrous and 15 g.L^−1^ agar. The strain was then denominated DMXS. In order to guarantee the purity of the isolated, the strain was inoculated in Petri plates containing growth media with pure D-Leu microcystin-LR. A pure isolate was considered a colony (single form) which was repeatedly replicated successfully.

### Evaluation of toxin [D-Leu^1^] microcystin-LR concentration by Elisa Immunoassay

Assessment of toxin levels was carried out by ELISA immunoassay with specific antibodies for microcystins. This test has high sensitivity and detects toxins in concentrations as low as 0.05 up to 2.5 μg.L^−1^. The ELISA kit (Abraxis, USA) was used in accordance with the methodology recommended by the manufacturer.

### Bacterial count

Bacterial count was performed through epifluorescence microscopy in a Zeiss Axioplan microscope (Germany) and using the software UTHSCSA Image Tool (Version 3.0) (http://compdent.uthscsa.edu/dig/download.html). Samples were filtered through polycarbonate membranes (Nuclepore, Whatman, UK; pore 0.2 μm) and colored with Irgalan black. Samples were then dyed with acridine orange in accordance with the methodology proposed by Hobbie *et al.* [26] to count bacteria in samples fixed with Lugol’s solution [27,28]. This fluorochrome emits green or orangish red fluorescence when it binds to bacterial DNA or RNA, respectively. We took advantage of the image by dividing the screen in several equal squares. For each screen containing an image divided in more than a 100 squares, we choose randomly a minimum of 30 squares to be counted.

### Molecular identification

DNA was extracted from 1-mL cell cultures by the Wizard Genomic DNA Purification kit (Promega, USA) in accordance with the supplier’s instructions. Extraction products were visualized on 1% agarose gel with GelRed (Biotium, USA). Primers of 16S segment (forward 5′-CCTACGGGAGGCAGCAG-3′ and reverse 5′-GACTACCAGGGTATCTAATC-3′) were designed as previously described [29]. DNA sample was amplified through polymerase chain reaction (PCR), which was performed in accordance with Ritchie *et al.* [29], except for the primer annealing temperature, which was optimized for 58°C. PCR products (approximately 400-bp long) were analyzed on GelRed-stained 1% agarose gel, with Low DNA Mass Ladder (Invitrogen, Thermo Fisher Scientific, USA) as the molecular weight marker, and then purified by enzymes exonuclease I and shrimp alkaline phosphatase. Purified PCR products were sequenced in both directions by a MegaBACE 1000 automated sequencer (GE Healthcare, UK). The resulting chromatograms were analyzed and the DNA sequence was identified through the Basic Local Alignment Search Tool (BLAST–GenBank National Center for Biotechnology Information).

The 16S rRNA gene sequences used in the phylogenetic analysis were retrieved from the GenBank (accession numbers given in parentheses) as follows: *P. aeruginosa* YL84 (CP007147), *P. alcaligenes* LMG 1224 T (Z76653), *P. citronellolis* DSM 50332 T (Z76659), *P. stutzeri* ATCC 17589 (U25432), *P. oleovorans* DSM 1045 T (Z76665), *P. flavescens* B62 (U01916) and *P. putida* DSM 291 T (Z76667), and were aligned through the ClustalX program [30]. An unrooted phylogenetic tree was constructed with the MEGA 6.0 program [31] and the statistical Neighbor-Joining method [32] with proportional (p) distance was used.

All experimental research reported in this manuscript was in compliance with the Brazilian Ethics Committee for Animal tests.

## Results

Samples of environmental water and sediment from the Patos Lagoon area including São Lourenço Beach were collected in order to isolate bacterial strains for the study of biodegradation of the hepatotoxin [D-Leu^1^] microcystin-LR. The analyses of water samples did not show meaningful differences except regarding the temperature; it was a slightly higher at spot P1 for several days.

Samples of water and sediment enabled the isolation of several strains (Table 1), whose ability for degrading microcystins were tested. However, most of them were not capable of degrading this cyanotoxin, except for the strain called DMXS.

**Figure 4:**
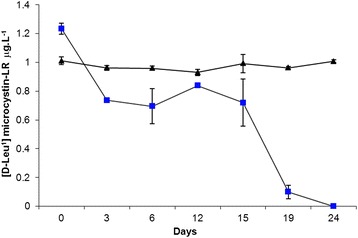
**Decrease in the concentration of toxin [D-Leu** ^**1**^ **] microcystin-LR by the bacterial strain DMXS. ▲ Control, ■ Treatment.**

The study of the biodegradation process of the hepatotoxin [D-Leu^1^] microcystin-LR was carried out in liquid medium. The decrease in the microcystin concentration in the medium is shown in Figure 4.

For the controls, since the samples had been autoclaved and the toxin concentration did not decrease during the experiment, no physical, chemical or biological degradation was observed. Therefore, the bacterial community was inactivated in the autoclave process.

In the treatment flask containing the DMXS strain, 40% of the total toxin concentration occurred in three days. After 15 days, the toxin concentration was kept stable, around 60%. From the 15th day on, there was a steep decrease: from 60% to 0 in only four days (Figure 4).

The rate of toxin decrease was calculated and *b* was 0.054579 or 5 μg L^−1^/day. Half-life was 12.7 days. The equation used for the half-life rate was developed in accordance with Ozawa *et al.* [33].

**Figure 5:**
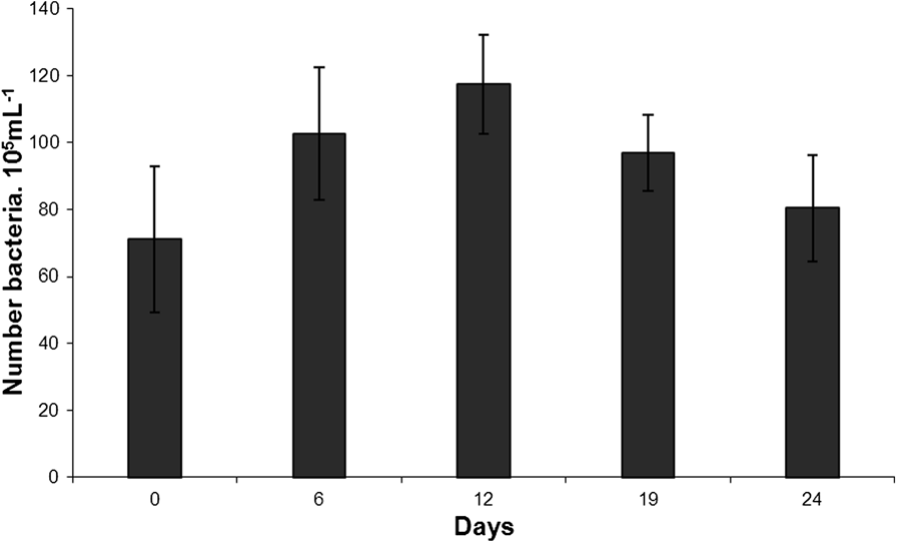
**Bacterial growth of the strain DMXS during the experimental period.**

Figure 5 shows the growth of the strain DMXS in parallel with the uptake of the toxin during the same period of the experiment. The bacterial concentration increased up to the 12th day. The decrease started on the 19th day, when the toxin concentration started to decrease concomitantly.

In the inoculum, the bacterial concentration was 71 × 10^5^ bacteria mL^−1^. It reached the highest concentration, 117 × 10^5^ bacteria mL^−1^, on the 12th day.

In the presence of the toxin, the strain DMXS grew slowly during the experiment. The decrease in the growth started after it had reached its highest concentration, 117 × 10^5^ bacteria.mL^−1^ when about 80% of the toxin had already been biotransformed.

**Figure 6:**
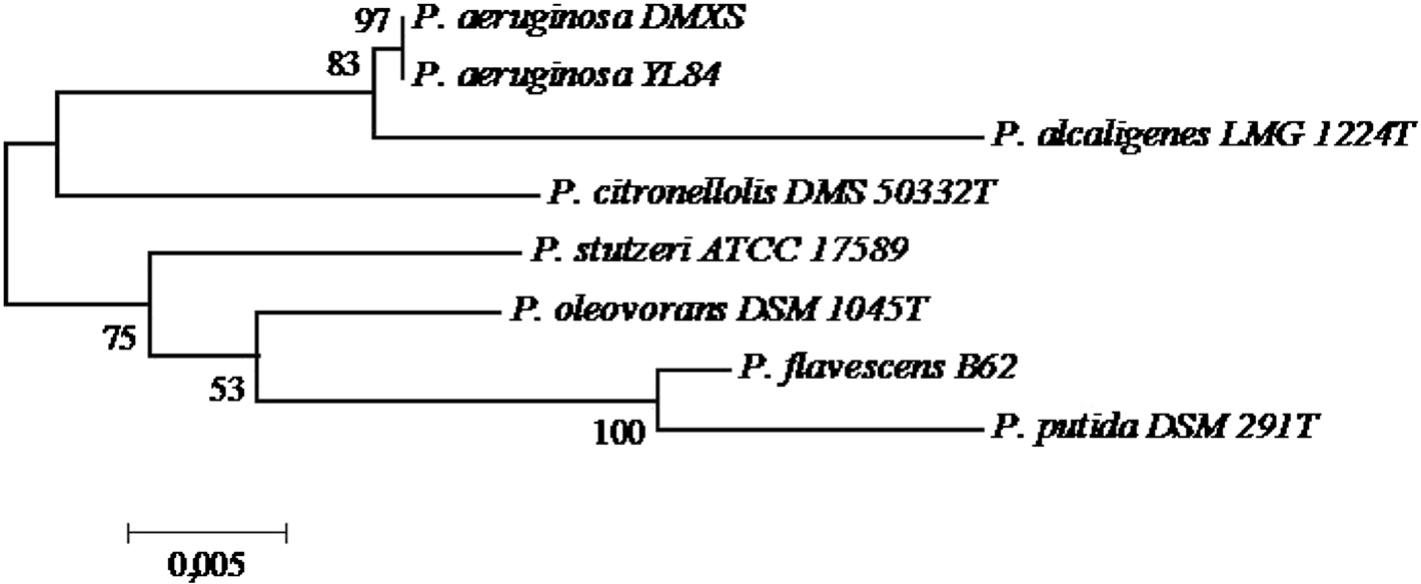
**Phylogenetic tree of the isolated DMXS and other species of the genus** ***Pseudomonas*** **based on 16S rDNA sequence analysis.**

The identification of the strain able of degrading the toxin was made by genetic sequencing; the total DNA was extracted from the isolated DMXS and the sequence of 16S rDNA was partially determined. The DNA sequence enabled the identification of the strain as *Pseudomonas aeruginosa* by the nucleotide BLAST tool (accession number HQ890467) and by the phylogenetic analysis using sequences from other species of the genus (Figure 6).

## Discussion

The waters in the hydrographic region of the Patos Lagoon have been studied and monitored regarding the presence and development of toxic cyanobacterial blooms as they are important for the economy and biodiversity in Rio Grande do Sul state, southern Brazil [10,12]. In an attempt to mitigate harmful effects of toxins on aquatic ecosystems, studies on the degradation and removal of cyanobacterial toxins have been carried out in several countries [11,16,17,34-37].

Biodegradation of the microcystin variant [D-Leu^1^] microcystin-LR was conducted in the laboratory using a strain isolated from sediment collected in the estuary. The strain DMXS was responsible for the decrease in the concentration of the toxin. It started right after the toxin was added to the experiment and was fast at the beginning. On the 19th day, toxin concentration was close to zero and, from the 24th day on, the toxin could not be identified anymore. The control was intact throughout the experiment; it means that the stability of the toxin was very high and that a possible contaminant bacterial community in the control was inactivated by the autoclave process.

The experiment was apparently stable for some time, from the 3rd to the 15th day, after the beginning of the toxin degradation process. However, degradation was slower only in this period. Other authors, including Chen *et al.* [18] and Edwards *et al.* [37], studied microcystin degradation and also found some similar results, i.g., some samples were only degraded after 10–20 days. The rate of toxin decrease was calculated and *b* was 0.054579 or 5 μg.L^−1^/day. Half-life was 12.7 days. Similar or higher values were also reported in other studies [18].

The organism responsible for the degradation of the toxin [D-Leu^1^] microcystin-LR is a bacterial strain isolated from sediment collected in the Patos Lagoon area (Rio Grande do Sul state, Brazil) at spot P1. Several studies of microcystin biodegradation have shown that there are bacteria in sediment collected in lakes [38-40]. This was observed both under aerobic [21,22] or anoxic conditions [40].

During the experiment, the bacterial strain DMXS grew slowly even at high temperatures, but having the toxin [D-Leu^1^] microcystin-LR as its single carbon source. Temperatures between 27°C and 29°C are considered high in comparison with mean temperatures in the place where the strain was isolated. This strain metabolism is low and appropriate to this kind of environment in which temperatures do not vary much. Although there is abundance of bacteria in this place, the specific cell activities of sediment bacteria is low [41].

It is also noteworthy that breaking the cyclic structure of microcystin molecules requires much energy from the bacterial metabolism. This may be a reason for the decrease on the rate of cell division and bacterial growth, as well.

The waters of Patos Lagoon harbor a vast diversity of bacteria [13]; however, as the present study has proven, the bacterial population capable of degrading cyanotoxins is small. The present findings agree with other studies that tested ability of bacteria from lake waters and sediment to degrade microcystins in the laboratory. Some have reported that only 17% of the strains are capable of carrying out the degradation [35]. An Argentinean study evaluated three isolated strains, but only one was capable of biodegrading microcystin-RR [42].

Studies on microcystin biodegradation conducted in China with samples collected in Taihu Lake showed that only 17 out of 96 water samples were capable of carrying out the biodegradation. Twelve out of these 17 samples were collected near the sediment-water interface [18].

The capacity of an aquatic bacterium to degrade microcystins is not common, since only few species have the proper metabolism needed to do it [43]. The genus *Sphingomonas* has been pointed by most studies [17,21,44-46], even though other genera are also capable of degrading these toxins [11,47,48].

Bacteria from sediment play an important role in the decomposition of organic matter and in geochemical cycles. These microorganisms are adapted to explore different sources of energy efficiently [6,49]. Hence, microbial degradation has been recommended as an effective process to eliminate microcystins in fresh water [18]. In this sense, the water column and the sediment play an important role in the degradation of microcystins in aquatic environments [18,35,37]. Therefore low levels of microcystins in an environment may be due to bacterial degradation [18].

Nowadays, biotechnological research on toxin biodegradation by sediment bacteria through removal of microcystins from water involves different techniques including: microorganisms immobilized in polyester resins, sand filters and bioreactors [22,36,50]. These biotechnological applications–employing different aquatic microorganisms that biodegrade microcystins–are considered an efficient process to remove such contaminants from water [50-52].

## Conclusion

The use of bacteria isolated from sediment for biotechnological applications is viable. Research has shown that sediment plays an important role as a source of bacteria capable of degrading cyanobacterial toxins. Results reported in this work corroborate previous studies and show that a bacterial strain from the genus *Pseudomonas aeruginosa* isolated from sediment collected in Patos Lagoon, in Brazil, biodegraded [D-Leu^1^] microcystin-LR in the laboratory.
